# Survivin and XIAP – two potential biological targets in follicular thyroid carcinoma

**DOI:** 10.1038/s41598-017-11426-3

**Published:** 2017-09-12

**Authors:** Thomas A. Werner, Levent Dizdar, Inga Nolten, Jasmin C. Riemer, Sabrina Mersch, Sina C. Schütte, Christiane Driemel, Pablo E. Verde, Katharina Raba, Stefan A. Topp, Matthias Schott, Wolfram T. Knoefel, Andreas Krieg

**Affiliations:** 1 Department of Surgery (A), Heinrich-Heine-University and University Hospital Duesseldorf, Moorenstr. 5, 40225 Duesseldorf, Germany; 2 Institute of Pathology, Heinrich-Heine-University and University Hospital Duesseldorf, Moorenstr. 5, 40225 Duesseldorf, Germany; 3 Coordination Centre for Clinical Trials, Heinrich-Heine-University and University Hospital Duesseldorf, Moorenstr. 5, 40225 Duesseldorf, Germany; 4 Institute for Transplantation Diagnostics and Cell Therapeutics, Heinrich-Heine-University and University Hospital Duesseldorf, Moorenstr. 5, 40225 Duesseldorf, Germany; 5 Division for Specific Endocrinology, Heinrich-Heine-University and University Hospital Duesseldorf, Moorenstr. 5, 40225 Duesseldorf, Germany

## Abstract

Follicular thyroid carcinoma’s (FTC) overall good prognosis deteriorates if the tumour fails to retain radioactive iodine. Therefore, new druggable targets are in high demand for this subset of patients. Here, we investigated the prognostic and biological role of survivin and XIAP in FTC. Survivin and XIAP expression was investigated in 44 FTC and corresponding non-neoplastic thyroid specimens using tissue microarrays. Inhibition of both inhibitor of apoptosis proteins (IAP) was induced by shRNAs or specific small molecule antagonists and functional changes were investigated *in vitro* and *in vivo*. Survivin and XIAP were solely expressed in FTC tissue. Survivin expression correlated with an advanced tumour stage and recurrent disease. In addition, survivin proved to be an independent negative prognostic marker. Survivin or XIAP knockdown caused a significant reduction in cell viability and proliferation, activated caspase3/7 and was associated with a reduced tumour growth *in vivo*. IAP-targeting compounds induced a decrease of cell viability, proliferation and cell cycle activity accompanied by an increase in apoptosis. Additionally, YM155 a small molecule inhibitor of survivin expression significantly inhibited tumour growth *in vivo*. Both IAPs demonstrate significant functional implications in the oncogenesis of FTCs and thus prove to be viable targets in patients with advanced FTC.

## Introduction

Malignant tumours of the thyroid gland are the most common endocrine neoplasms in men with the differentiated carcinomas (DTC) accounting for approximately 90% of all of them^1^. Follicular thyroid carcinomas (FTC) represent a subgroup of DTCs, which originate from the follicular cells of the thyroid gland^2^. The adequate therapy upon establishing the diagnosis remains the total thyroidectomy with postoperative radioiodine therapy^1, 3^. Interestingly, even with distant metastases the overall survival rate remains approximately 50% over five years if the tumour is positive for radioactive iodine (RAI). However, this rate deteriorates to mere 19% once the tumour fails to adequately retain RAI^4^.

Large cohort studies revealed that approximately one third of all patients with DTC and distant metastasis lack RAI uptake at the time of diagnosis^5–7^. In addition, RAI positive tumours may turn RAI negative during the course of disease, reducing the therapeutic options as well as overall survival time considerably^5, 8, 9^.

Due to the large amount of patients that will turn RAI refractory over their course of the disease, novel adjuvant therapeutic strategies have been in the focus of researchers worldwide^4^. This new appreciation of RAI non-avid FTCs has led to the replacement of doxorubicin as standard of care by multi-kinase inhibitor sorafenib. In this context, sorafenib prolonged progression free survival^10, 11^ but did not improve the poor 10-year overall survival rate of patients with metastasized RAI refractory FTC^5, 11^.

Therefore, new druggable therapeutic targets are in high demand to lighten the bleak prognosis in this subset of patients. One of the hallmark features of carcinoma cells is its inherent strife for survival and its consequently altered regulation of apoptosis and proliferation^12, 13^. In this regard, a group of anti-apoptotic proteins known as the Inhibitor of apoptosis protein (IAP) family has gained considerable attention^14^. They act through protein-protein interaction and influence cell death, mitosis, migration, metastases and inflammation^15, 16^. The two most renowned IAPs are survivin/BIRC5 and X-linked inhibitor of apoptosis protein XIAP/BIRC4. Synergistically they inhibit apoptosis by targeting caspase-3 and -9^17^ and induce resistance to conventional chemotherapeutic agents^18^. In addition, XIAP targets the Ripoptosome^19^, whereas survivin is a key regulator of cell cycle progression and proliferation^20^. Together, survivin and XIAP induce the nuclear translocation of Nuclear Factor kappaB (NF-kB), supporting tumour cell invasion and metastasis^16, 21^.

Unsurprisingly, great efforts have been made to design specific small molecule IAP-antagonists, to induce programmed cell death. The most successful survivin targeting agents have been YM155 (Sepantronium bromide) and tetra-O-methyl nordihydroguaiaretic acid (M4N; Terameprecol; EM-1421), with both demonstrating anti-tumour activity in several phase I and II studies^22–25^. Concerning XIAP, small-molecule mimetics that imitate endogenous IAP inhibitor second mitochondria derived activator of caspase (Smac), have also been successfully tested in preclinical phase I studies^26^.

Although a small number of studies has examined the expression of IAPs in thyroid malignancies in general, none of these studies have further discriminated between the stage dependent expression and prognostic relevance of survivin and XIAP in FTC^27–29^. In addition, to date no data exists about their functional implications in follicular thyroid malignancies. As a result, the actual biological role of survivin and XIAP in FTC remains largely unknown. The aim of our study was to fill this gap and to evaluate their prognostic and therapeutic potential in FTCs.

## Results

### Expression of survivin and XIAP in FTC

A total of 44 patients were included in our analysis. The clinicopathological characteristics are summarized in Supplementary Table 1. Ten patients were lost to follow up and could not be included in our survival analysis. Median overall survival was 47 months (range 0–197 months).

**Figure 1:**
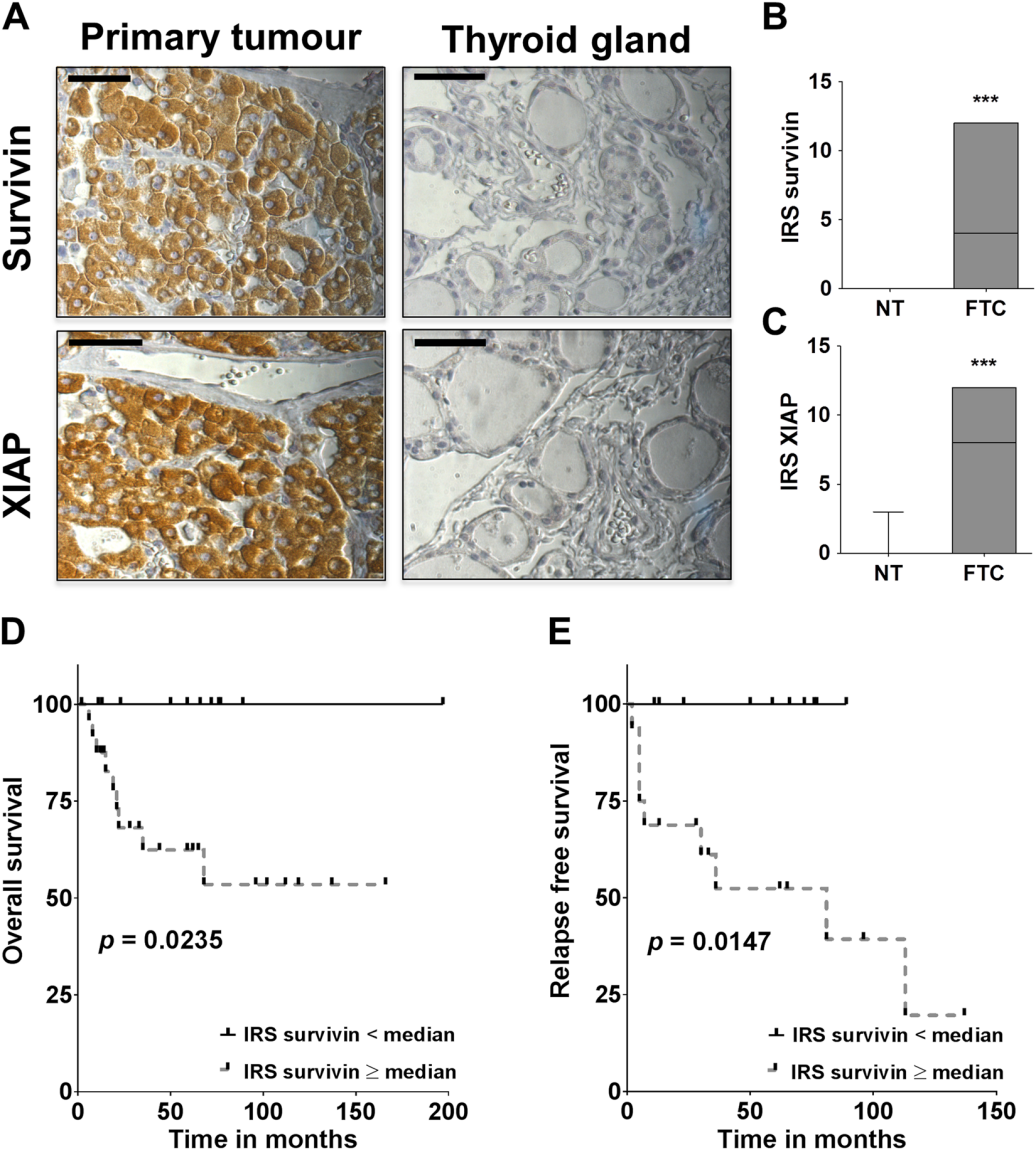
Expression of survivin and XIAP in FTC. (**A**) Representative images of FTC specimens (left) and corresponding non-neoplastic thyroid tissue (right) that were immunohistochemically stained using antibodies raised against human survivin or XIAP. Images were captured at 400x magnification and scale bar indicates 50 µm. (**B** and **C**) Boxplots display the median survivin and XIAP IRS with the maximum and minimum for FTC and corresponding non-neoplastic thyroid tissue (NT). (**D** and **E**) Kaplan–Meier curves for overall and relapse free survival based on survivin expression levels, categorized according to the IRS into high (IRS survivin ≥ median) and low (IRS survivin < median). Numerical data were analysed using the two-tailed nonparametric Mann-Whitney U test (***p < 0.001).

Survivin and XIAP were only expressed in FTC tissues whereas the corresponding non-neoplastic thyroid tissue specimens stained negatively for both IAPs (survivin: median: 4; range: 0–12; *p* < 0.0001, XIAP: median: 8; range 0–12; *p* < 0.0001; Fig. 1A–C). In addition, we found significantly higher expression levels of both IAPs in FTCs, when compared to follicular thyroid adenomas (data not shown). A correlation between XIAP expression and clinicopathological parameters became not evident. Survivin on the other hand exhibited a significantly higher expression in T3/4 tumours when compared to T1/2 tumours (Supplementary Table 2).

To explore the prognostic value of survivin and XIAP, we performed univariate and multivariate survival analyses including variables such as age, gender, tumour size, UICC stage as well as survivin and XIAP expression levels according to the IRS. Univariate analysis revealed that high survivin expression was associated with a significantly reduced overall survival (*p* = 0.0235; Fig. 1D, Table 1). This observation was confirmed in multivariate analysis with all available variables. In this context, survivin proved to be an independent negative prognostic marker in FTC (HR 1.558; 95% CI: 1.099–2.207; *p* = 0.013; Table 1).

**Table 1:** Overall survival - univariate analysis.

Variables	HR	CI (lower - upper 95%)	*p-*value	
Age	2.225	0.644–7.694	0.206	
Sex	0.485	0.130–1.808	0.281	
Tumour size [cm]	1.623	0.379–6.944	0.426	
UICC I/II vs. UICC III/IV	2.503	0.67–9.358	0.173	
XIAP expression	0.781	0.134–4.540	0.783	
Survivin expression	4.822	1.236–18.82	0.024	*
**Overall survival - multivariate analysis**				
Age	1.075	1.002–1.152	0.043	*
Sex	0.208	0.026–1.685	0.141	
Tumour size [cm]	1.581	0.964–2.591	0.069	
UICC I/II vs. UICC III/IV	17.62	0.537–578.126	0.107	
XIAP expression	0.853	0.674–1.080	0.188	
Survivin expression	1.558	1.099–2.207	0.013	*

Abbreviations: CI = confidence interval; HR = hazard ratio; XIAP = X-linked inhibitor of apoptosis protein; UICC = Union internationale contre le cancer; *p < 0.05.

Interestingly, Kaplan-Meier curves for relapse free survival demonstrated that primary tumours exhibiting high survivin expression levels had a higher risk of early recurrence (*p* = 0.0147; Fig. 1E). However, due to the small number of patients with recurrent disease, a multivariate analysis was not feasible.

### Survivn and XIAP knockdown inhibits proliferation and tumour growth

**Figure 2:**
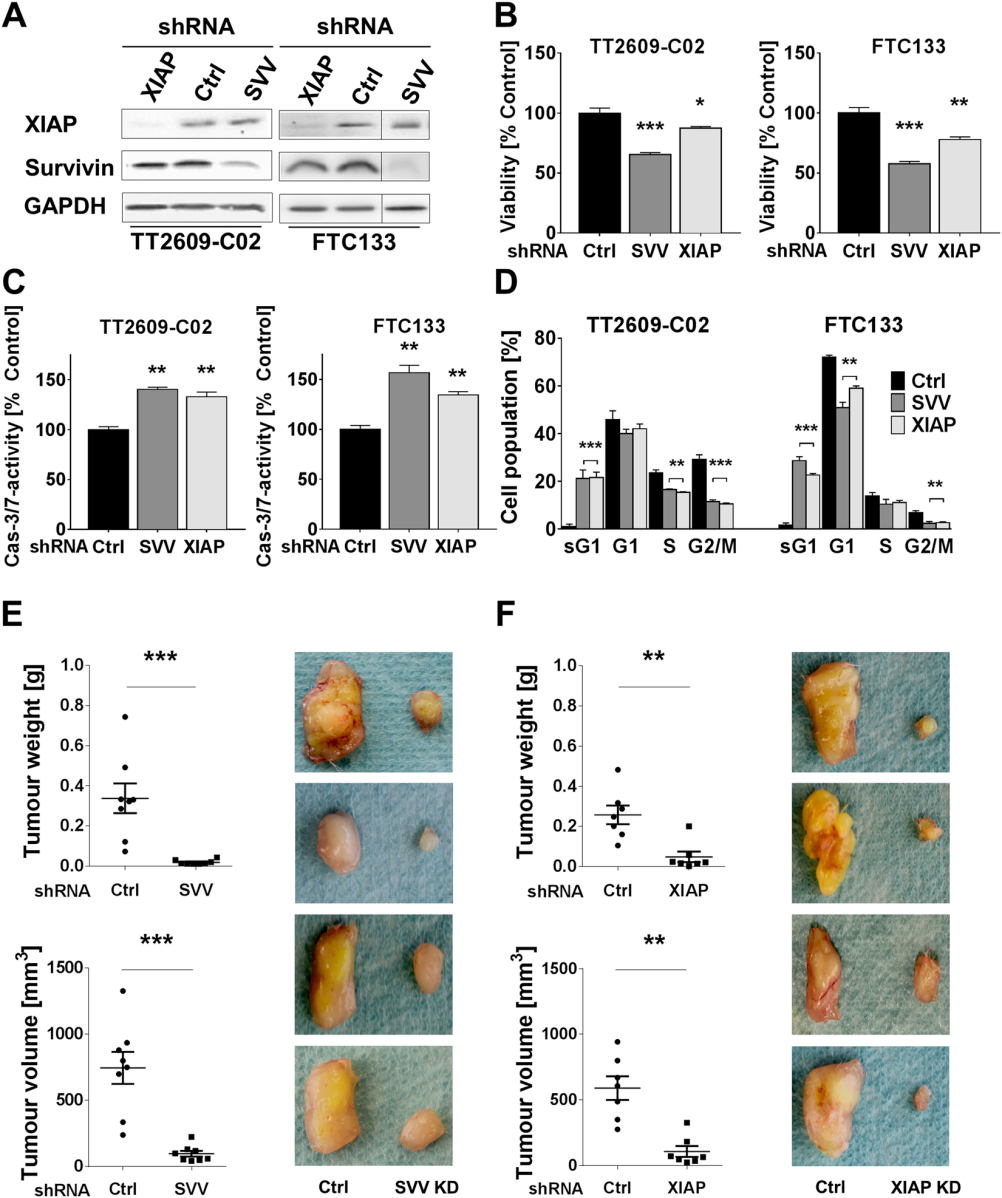
Survivin and XIAP knockdown compromises FTC cell growth *in vitro* and *in vivo*. (**A**) Gene-specific shRNA knockdown of survivin (SVV KD) and XIAP (XIAP KD) for both FTC cell lines TT2609-C02 and FTC133 was confirmed using western blot analysis with GAPDH serving as loading control. Non-specific shRNA served as control (Ctrl). Blots are cropped to improve clarity. Black lines are inserted where lanes were not directly adjacent on the original blot. The full-length blots are presented in Supplementary Fig. 3a and b. The functional implications of a survivin or XIAP knockdown on (**B**) cell viability and (**C**) caspase-3/7-activity were investigated. (**D**) SVV KD and XIAP KD resulted in significantly altered cell cycle phases as demonstrated by PI staining and FACS. Cells are grouped according to the respective cell cycle phase (sG1 = subG1) and populations are displayed as percentages. All values are expressed in means + SEM of at least three independent experiments. Statistical significance was calculated by two-tailed nonparametric Mann-Whitney test (*p < 0.05; **p < 0.01; ***p < 0.001). *In vivo* changes in tumour volume and weight caused by (**E**) survivin or (**F**) XIAP knockdown were investigated using a xenograft mouse model. TT2609-C02 knockdown cells were subcutaneously injected into the left flank region of 6–8-week-old NOD-Scid IL2rgamma^null^ mice. FTC cells transduced with non-silencing shRNA (Ctrl) were injected into the other side to serve as control. Images display representative tumour samples of the respective gene specific knockdown cell lines (SVV, XIAP) and non-silencing control (Ctrl). Box plots represent the mean ± SEM. Numeric data were analysed by Wilcoxon matched pairs test (**p < 0.01; ***p < 0.001).

To elucidate the biological role of survivin and XIAP in FTC, we induced a shRNA-specific knockdown in the two FTC cell lines TT2609-C02 and FTC133. The knockdown was verified on protein levels (Fig. 2A). A non-targeting lentiviral shRNA construct served as negative control.

Next, we quantified cell viability and proliferation in freshly transduced knockdown cells by using MTS and BrdU assays. Both, survivin as well as XIAP knockdown resulted in a significant reduction of cell viability and proliferation in both cell lines (Fig. 2B and Supplementary Fig. 1a and b).

To investigate the implication of the gene-specific knockdown on apoptosis, we measured caspase-3 and -7 activities. Survivin and XIAP knockdown cell lines demonstrated a significant increase in caspase-3/7 activation when compared to cells transduced with non-specific shRNA (Fig. 2C).

In addition, cell cycle analysis revealed significant alterations in gene-specific knockdown cell lines for survivin (SVV KD) and XAIP (XIAP KD, Fig. 2D). Both knockdown cell lines demonstrated for both IAP knockdowns a significant reduction of cells in the G2/M phase (TT2609-C02: SVV KD: 11.5% vs. 30.1%, p < 0.001; XIAP KD: 10.4% vs. 30.1%, p < 0.001; FTC133: SVV KD: 2.6% vs. 13.9%, p < 0.01; XIAP KD: 2.45% vs. 13.9%, p < 0.01), whilst both cell lines also exhibited significant sub-G1 cell populations. Whereas the G1 phase remained unaffected for the TT2609-C02 knockdowns, both survivin and XIAP FTC133 knockdown cells showed a significant reduction of cells in this phase (SVV KD: 53.8% vs. 72.1%, p < 0.01; XIAP KD: 59.8% vs. 72.1%, p < 0.01). TT2609-C02 knockdown cell on the other hand demonstrated a significant reduction of cells in the S phase when compared to their control transfected counterparts (SVV KD: 14.4% vs. 24.8%, *p* < 0.01; XIAP KD: 16.2% vs. 24.8%, *p* < 0.01).

Next, we examined the functional aspects of survivin or XIAP knockdown cells *in vivo*, using a xenograft mouse model. In accordance with our *in vitro* data, survivin knockdown impaired the tumour-forming ability of TT2609-C02 cells characterized by a reduced tumour volume (Ctrl: 746.1 ± 320.2 mm^3^ vs. SVV KD: 98 ± 57.3 mm^3^; *p* < 0.001) and tumour weight (Ctrl: 0.337 ± 0.196 g vs. SVV KD: 0.019 ± 0.011 g; *p* < 0.001) when compared to control transfected cells (Fig. 2E). XIAP-deficient cells exhibited comparable results with a significantly reduced tumour volume (Ctrl: 590.8 ± 220.7 mm^3^ vs. XIAP KD: 107.9 ± 101.8 mm^3^; p < 0.01) and weight (Ctrl: 0.26 ± 0.11 g vs. XIAP KD: 0.048 ± 0.064 g; p < 0.01; Fig. 2F).

### Small molecule antagonists against survivin and XIAP

After establishing the functional significance of survivin and XIAP in FTC, we next investigated the implication of a chemical inhibition of survivin and XIAP. Therefore, we treated wild type FTC cell lines TT2609-C02 and FTC133 with increasing concentrations of survivin small molecule antagonists YM155 or M4N and XIAP-antagonizing Smac mimetic AT406, respectively.

**Figure 3:**
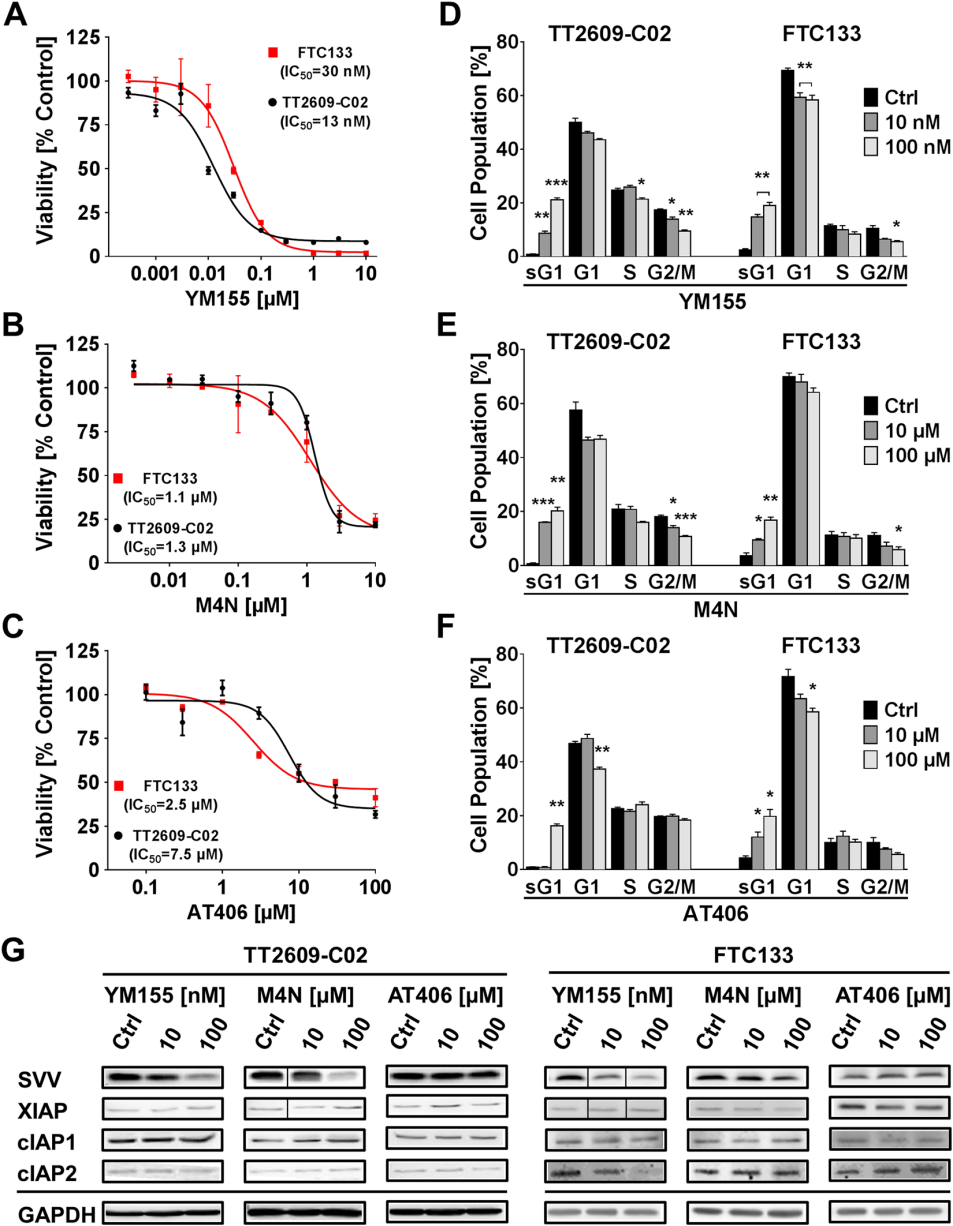
Effect of small molecule survivin or XIAP inhibitors on viability and the cell cycle of FTC cells. FTC cells were treated with increasing concentrations of YM155 (**A** and **D**), M4N (**B** and **E**) and AT406 (**C** and **F**). Changes in cell viability (**A**–**C**) are illustrated in a logarithmic fashion to demonstrate the dose dependent effect of the respective compounds. IC_50_ values represent the mean 50% inhibitory concentration. (**D**–**F**) Cell cycle analyses were performed by using FACS and PI staining. Cell populations are grouped according to the distinct cell cycle phase (sG1 = subG1). (**G**) The effect of increasing concentrations of YM155, M4N or AT406 on protein expression levels of IAP family members survivin, XIAP, cIAP1 and cIAP2 was evaluated by western blot analysis. GAPDH served as loading control. Blots are cropped to improve clarity. Black lines are inserted where lanes were not directly adjacent on the original blot. The full-length blots are presented in Supplementary Fig. 3c–f. All experiments were repeated at least three times. Values are expressed in means + SEM. Numeric data analysis was carried out using the two-tailed nonparametric Mann-Whitney U test (*p < 0.05; **p < 0.01; ***p < 0.001).

All of these compounds induced a dose dependent decrease in cell viability with an IC_50_ of 13 nM, 1.3 µM and 7.5 µM for YM155, M4N and AT406 in the TT2609-C02 cell line, whilst the FTC133 cell line showed itself slightly more susceptible to the small molecule antagonists with IC_50_ values of 30 nM, 1.1 µM and 2.5 µM for YM155, M4N and AT406, respectively (Fig. 3A–C). In line with these results, we observed a significantly reduced cell proliferation in TT2609-C02 and FTC133 cells treated with YM155 and M4N (Supplementary Fig. 2a and b). However, for AT406 this effect only became evident in the TT2609-C02 cell line at micromolar concentrations (Supplementary Fig. 2c). To further elucidate the functional implications of these compounds on the cell cycle, we performed FACS analysis using PI DNA staining (Fig. 3D–F). For all compounds we found a significant increase in the subG1 population. However, the effect of AT406 on the TT2609-C02 cell line only became obvious at a very high concentration of 100 µM (Ctrl: 0.83% vs. 100 µM: 16.2%; *p* < 0.01). Again, FTC133 proved to be more susceptible to AT406 treatment (Ctrl: 4.3% vs. 10 µM: 15.4%; p < 0.05). In addition, survivin targeting compounds YM155 and M4N induced a decrease of cells in the G2/M phase.

Of note, both transcriptional inhibitors YM155 and M4N reduced survivin protein levels in a concentration-dependent manner (Fig. 3G). In contrast, the expression of other IAPs including cIAP1, cIAP2 and XIAP remained unchanged.

**Figure 4:**
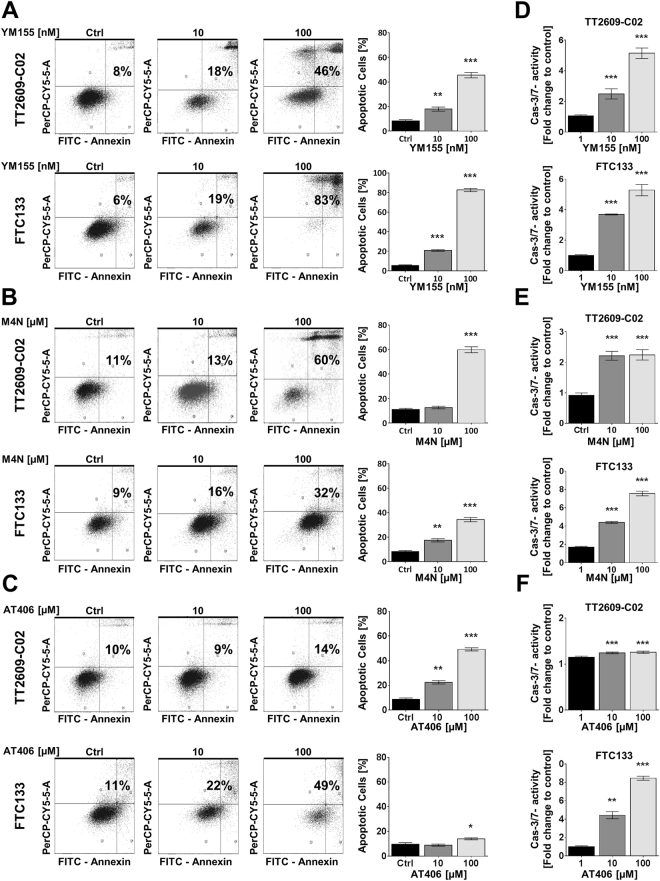
Apoptosis-inducing capacity of IAP antagonists YM155, M4N and AT406 in FTC cells. (**A**–**C**) Annexin-V/PI staining and FACS analyses of FTC cells demonstrated a dose dependent increase of annexin positive apoptotic cells when incubated with YM155, M4N and AT406 for both FTC cell lines TT2609-C02 and FTC133 (Ctrl = vehicle control). (**D** and **E**) Survivin antagonists YM155 and M4N induced a dose dependent activation of caspase-3/7. (**F**) Smac mimetic AT406 exhibited less potent caspase activation, with FTC133 proving the more susceptible cell line. Results are expressed as fold change to vehicle control. Statistical significance was calculated by two-tailed nonparametric Mann-Whitney test (*p < 0.05; **p < 0.01; ***p < 0.001).

To further investigate the ability of survivin and XIAP small molecule antagonists to induce apoptotic cell death, we performed FACS analysis using Annexin V/PI staining (Fig. 4A–C). Again, these experiments revealed a stronger effect of survivin antagonists on FTC cells when compared to the XIAP-antagonizing Smac mimetic AT406. In line with this observation, caspase-3/7-activity was markedly elevated in FTC cells incubated with YM155 and M4N. The effect of AT406 was less profound (Fig. 4D–F).

### YM155 impairs tumour growth *in vivo*

Since our *in vitro* data demonstrated that treatment with survivin antagonists most effectively killed FTC cells, we decided to confirm these observations in a xenograft mouse model. We concentrated on survivin antagonist YM155 because it exhibited the strongest anti-tumour effects and used the FTC cell line TT2609-C02 for subcutaneous tumour cell injection. After 28 days of well-tolerated continuous treatment without adverse effect or drop out, the experiment was terminated.

**Figure 5:**
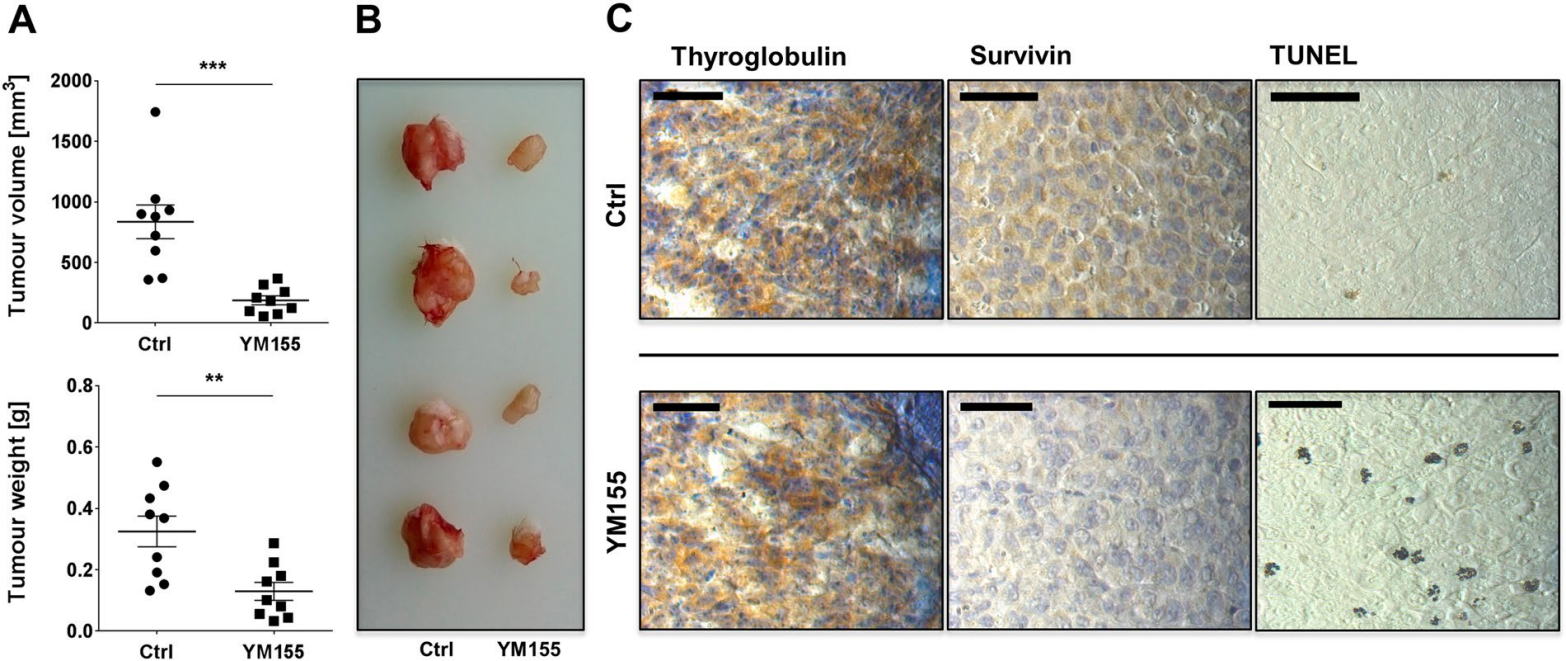
Survivin targeting compound YM155 impairs tumour growth *in vivo*. NOD-Scid IL2rgamma^null^ mice bearing TT2609-C02 xenografts were treated by daily intraperitoneal injection of YM155 (3 mg/kg) or vehicle control (Ctrl). (**A**) Survivin antagonist YM155 significantly impaired tumour volume and weight (**p < 0.01; ***p < 0.001). (**B**) Four representative tumour samples from the treatment and control group demonstrate the growth inhibitory effect of YM155. (**C**) Immunohistochemical staining of FFPE sections obtained from FTC xenografts confirmed a decrease in survivin protein levels accompanied by an increase of TUNEL-positive cells only in the treatment group. Thyreoglobulin remained unaffected in both therapeutic arms. Images were captured at 400x magnification and scale bar indicates 50 µm.

Treatment with YM155 suppressed *in vivo* tumour growth, which was reflected by a median volume of 879.03 ± 392.73 mm^3^ for the control group compared to 182.25 ± 103.79 mm^3^ for the treatment group (Fig. 5A and B; *p* < 0.001). Moreover, the median tumour weights at study termination were 0.368 ± 0.142 g and 0.101 ± 0.083 g for the control and YM155 treatment group, respectively. Importantly, both male and female mice exhibited the same responsiveness.

Next, we performed immunohistochemical staining of paraffin embedded tumour sections from each experimental arm. As expected, tumour specimens from the YM155 treatment group showed a marked reduction of survivin protein expression, accompanied by an increase of apoptotic tumours cells as demonstrated by TUNEL assay (Fig. 5C). The expression of thyroglobulin remained unchanged in specimens from both experimental arms.

## Discussion

FTC is the second most common malignancy of the thyroid gland. FTC’s generally favourable outcome is mostly due to its excellent surgical and adjuvant treatment regimes. While surgical radicalness differs depending on the extend of tumour invasion, the postoperative therapeutic protocols are mainly based on RAI treatment^1, 3^. However, if a tumour is primarily RAI refractory or turns refractory during the course of disease, the patient’s prognosis will be negatively impaired with only limited options for a second line therapy^8^.

In this context, inability of carcinoma cells to undergo apoptotic cell death marks a hallmark feature of oncogenesis. A group of proteins that modulate the balance between proliferation and apoptosis are the IAP-family members. They have been linked to resistance mechanisms to conventional chemotherapeutics and their prognostic power has been outlined in multiple tumour entities^18^.

To date, only a small number of studies have investigated the expression of survivin and XIAP in follicular thyroid malignancies. In the study by Chen *et al*., DTC and undifferentiated thyroid carcinomas were grouped together as one and the general survivin expression in this combined group was compared to non-neoplastic thyroid tissue specimens. No subsequent functional analyses on the inhibitory effect of survivin were performed^27^. Likewise, Waligórska-Stachura and colleagues only determined the expression level of survivin in different thyroid malignancies including papillary, follicular, medullary and undifferentiated thyroid carcinomas. In the following analyses however, they grouped the different tumour entities as one and compared them to non-neoplastic thyroid tissue^28^.

Also for XIAP, up until now no scientific data regarding its stage dependent expression in FTC exists. Xiao *et al*. investigated the expression of XIAP in different histological types of thyroid carcinomas and other non-neoplastic thyroid disorders^29^. Again, only a small set of 6 patients with FTC was immunohistochemically investigated for IAP expression and no FTC-specific subsequent analyses regarding association with clinicopathological parameters or overall survival was performed. Notably however, all samples stained negative for XIAP. This may be attributed to the small sample size and different antibody used as the vast majority of our FTC tissue specimens stained positive for XIAP. In a study published by Yim *et al*. 25% of the 8 investigated FTC tissue specimens stained positive for XIAP^30^. However, no subsequent analyses apart from the actual quantification of expression were performed. Taken together, these publications illustrate the current shortage of resilient clinical and functional data regarding the role of IAPs in FTCs. To our knowledge, our study is the first to investigate the stage dependent expression and prognostic relevance of survivin and XIAP in FTCs and which further stratifies their functionality both *in vitro* as well as *in vivo*. In our cohort, both survivin and XIAP were uniquely expressed in carcinoma tissue, when compared to non-neoplastic thyroid tissue specimens. Interestingly, only survivin exhibited a strong correlation with tumour size and proved to be an independent negative prognostic marker. In addition, survivin expression was also negatively associated with relapse free survival. Of note, our results are in line with recently published data on the prognostic relevance of IAPs in medullary thyroid^31^, gastric and colorectal carcinoma^21, 32^, underscoring the role of these two IAPs in oncogensis^15^.

Importantly, our functional experiments further confirmed the biological implications of an inhibition of IAP expression in FTC. We could demonstrate for both survivin and XIAP that a shRNA induced knockdown not only significantly increased caspase-3/7-activity but also markedly reduced proliferation and viability. This was further associated with a marked reduction of cells in the G2/M-phase as well as the induction of a subG1 population. These changes cumulate in the significant reduction of tumour volumes and weights *in vivo*.

It is well known that IAPs are much more than just inhibitors of apoptosis, influencing cellular proliferation, signalling and differentiation^33^. Mechanistically, the reduced expression of survivin and XIAP prohibits the inhibition of caspases^17^ and induces the forming of the Ripoptosome, a ~2 MDa large cell death-inducing platform which mediates apoptosis and necroptosis in response to genotoxic stress^19^. Furthermore, survivin changes the forging of the chromosomal passenger complex, a key regulator of cell cycle progression^20^, enabling the way for apoptosis and cell cycle arrest. XIAP on the other hand regulates cell cycle progression through inhibition of cyclin D1 via its ubiquitin ligase E3, repressing anchorage-independent cell growth^34^.

Even though the treatment with Smac mimetic AT406 caused a dose dependent reduction of cell viability and a significant increase in caspase-3/7-activity the results across both cell lines were less profound than in the XIAP knockdown cells. Smac mimetics unfold their apoptosis-inducing capacity through direct binding of XIAP, preventing its interaction with caspase-3, -7, and -9^26, 35^. Unfortunately, a low response rate to Smac mimetics is not an uncommon feature. This can in part be explained by different resistance mechanisms in malignant cells. The up-regulation of TNFα mediates the induction of cIAP2 via NF-κB, which in turn counteracts inhibitory effects of AT406^36^. Other mechanisms have been reported regarding the inability to form a ripoptosome or a defective PI3K signalling pathway^36, 37^.

Regarding survivin on the other hand, a targeted approach with antagonizing small molecules YM155 and M4N demonstrated dose dependent functional effects that were in line with our knockdown experiments. Whilst both compounds work as transcriptional repressors, YM155 directly binds to the Sp1-binding site of the survivin promoter, thus inhibiting survivin expressions specifically^38^. M4N on the other hand is not survivin gene-specific, but unfolds its potential by binding the transcription factor Sp1^23, 39, 40^. These different points of attack may account for the higher effectiveness of YM155 and further underscores the specific role of survivin in FTC.

Despite first encouraging results of multi-kinase inhibitor sorafenib, the prognosis of patients with RAI refractory FTC remains poor and their clinical management is an on-going challenge^11, 41^. With no effective therapeutic option at hand, our experimental data offer a promising new approach for the treatment of RAI non-avid FTC that warrants further investigation and holds the potential for future clinical applications. This new valuable insight into the oncogenesis of FTC, needs however to be further substantiated in pre-clinical trials, where survivin and XIAP have to prove their worth as a new viable option in FTC treatment.

## Material and Methods

### Patient selection and clinicopathological data

All patients with histologically confirmed FTC who underwent curative surgery at the Department of Surgery (A), University Hospital Duesseldorf between 1992 and 2014 were retrospectively reviewed. Two independent and experienced pathologists confirmed the histological diagnosis for each tissue specimen. FTC was defined as a thyroid carcinoma with follicular differentiation in the absence of papillary nuclear features and either capsular or vascular invasion. Our exclusion criteria comprised insufficient pathological reports, missing clinical data, incomplete resection of the tumour specimen and insufficient tumour material for subsequent analysis. Data regarding overall survival as well as clinical parameters including age at first diagnosis, gender, date and type of surgery, affected lobe and TNM stage were obtained from our prospectively maintained clinical database. All investigated tumour specimens were staged according to the 7^th^ edition of the UICC classification^42^. The study was carried out in accordance to Good Clinical Practice, the Declaration of Helsinki and local rules as well as regulations of the country. An approval from the institutional ethics committee of the Medical Faculty, Heinrich Heine University Duesseldorf was obtained, which functioned as an informed consent statement (reference number: 3821).

### Tissue microarray and immunohistochemistry

All formalin-fixed paraffin-embedded tissue specimens were collected from the Institute of Pathology, University Hospital Duesseldorf. The construction of the tissue microarray (TMA), immunohistochemistry and analysis of protein expression using the immunoreactivity score (IRS) reported by Remmele^43^ were performed as described previously^31^. For each patient two representative tissue cylinders of the primary tumour, one tissue core of the corresponding non-neoplastic thyroid gland and two tissue samples of a lymph node or distant metastasis if present were taken. FTC tissue cores were taken from the centre of the malignant lesion, whereas the corresponding non-neoplastic tissue specimens were taken from unaffected adjacent tissue or, where available from the opposite lobe. The respective cylinders had a diameter of 1.0 mm.

For immunohistochemical staining the following primary antibodies were used: rabbit polyclonal anti-survivin (NB500–201; 1:750 dilution; Novus, Littleton, CO, USA), mouse monoclonal anti-XIAP (Clone 48; 1:50 dilution; BD Biosciences, San Jose, CA, USA), rabbit polyclonal anti-human thyroglobulin (A0251; 1:4000 dilution; Dako, Glostrup, Denmark). Isotype control was performed using mouse IgG1k (MOPC-21; 1:50 dilution; Abcam, Cambridge, UK) and rabbit immunoglobulin fraction (X0903; 1:1000 dilution; Dako, Glostrup, Denmark). The prognostic power of the respective marker was assessed according to the REporting recommendations for tumour MARKer prognostic studies (REMARK)^44^.

### Cell line and lentiviral transduction

Human FTC cell line TT2609-C02 was purchased from the Leibniz Institute DSMZ-German Collection of Microorganisms and Cell Cultures (DSMZ no.: ACC 510). The cell line originated from local tumour recurrence of a secondary RAI refractory FTC. Under culture conditions the cell line demonstrates no relevant ^125^I uptake^45^.

The human FTC cell line FTC133 was purchased from Sigma-Aldrich (catalogue no.: 94060901). The cell line was obtained from a patient with metastatic FTC and does not demonstrate iodine uptake under culture conditions^46, 47^. Authenticity of both cell lines was confirmed by Short Tandem Repeat (STR) DNA profiling.

For lentiviral transduction 2.5 × 10^6^ HEK293FT cells were incubated in DMEM/10% FCS over night at 37 °C and 5% CO_2_. Transfection was performed using Lipofectamine2000^TM^ with envelope plasmid pCMV-VSVG, packaging plasmid psPAX2 (both Addgene, Cambridge, MA, USA) and respective targeting vectors for XIAP (clone V2LHS-94578; mature antisense sequence: TTACAAGTGACTAGATGTC), survivin (clone V2LHS_262484; mature antisense sequence: TTCCTAAGACATTGCTAAG) or GIPZ non-silencing Lentiviral shRNA (all Open Biosystems, Dharmacon, Lafayette, CO, USA).

Lentiviral supernatant was harvested and FTC cell lines transduced using polybrene. Knockdown was validated by western blot analysis.

### Functional *in vitro* assays

Cell viability and proliferation were assessed in 96-well culture plates with cells plated at a concentration of 4 × 10^3^ per well. After 24 h cells were incubated for 48 h with YM155, M4N, AT406 or vehicle control at equimolar concentrations. To assess cell viability and proliferation, CellTiter 96® AQueous Non-Radioactive Cell Proliferation Assay (Promega, Madison, WI, USA) at an absorbance at 490 nm or Cell Proliferation ELISA, BrdU assay (Roche Applied Science, Mannheim, Germany) at an absorbance at 370 nm were performed according to the manufacturer’s protocol. All assays were analyzed using an Infinite® 200 microplate reader (Tecan Group Ltd., Crailsheim, Germany).

For cell cycle analysis, FTC cell lines were harvested, washed in ice cold PBS and resuspended in 80% ethanol. After incubation for 2 hours, cells were washed in PBS and RNAse A (100 µg/ml) together with propidium iodide (PI; 50 µg/ml) was added. Finally, cells were analyzed by Fluorescence-activated cell sorting (FACS) after 30 minutes of incubation at 37 °C using a BD FACSCanto™ II (BD Biosciences, San Jose, CA, USA).

Apoptotic cell death was quantified by using the FITC Annexin V/Dead Cell Apoptosis Kit (Molecular Probes, Eugene, OR, USA) and Caspase-Glo® 3/7 Assay (Promega Corp, Mannheim, Germany) according to the manufacturer’s protocol.

### Xenograft mouse model

To evaluate the *in vivo* effect of a stable survivin or XIAP knockdown on tumour growth in a xenograft mouse model, 1 × 10^6^ TT2609-C02 gene specific knockdown cells were dissolved in 200 µl sterile Matrigel/PBS solution and subcutaneously injected into the left flank of 6–8-week-old NOD-Scid IL2rgamma^null^ mouse. Simultaneously, TT2609-C02 cells transduced with unspecific shRNA were subcutaneously injected into the right flank of the same mouse. Accordingly, each mouse could serve as its own control. The mice were sacrificed 7 weeks after the beginning of the experiment.

To evaluate the growth inhibitory effect of a pharmacological inhibition of survivin, 1 × 10^6^ TT2609-C02 cells were dissolved in a sterile Matrigel/PBS suspension and subcutaneously injected into the left flank of 6–8-week-old NOD-Scid IL2rgamma^null^ mice. After the tumour volume reached 100–200 mm^3^ in a two-dimensional caliper measurement (v = (l × w^2^/2) [v = volume (mm^3^)]; l = length (mm), w = width (mm)), the animals were separated into a treatment and control group. The animals were paired with regards to their tumour volume to ensure conformity and comparability between the two groups. Afterwards, animals were treated with 3 mg/kg YM155 by a daily intraperitoneal injection in the treatment group or the equivalent amount of sterile saline as vehicle solution in the control group. After four weeks the mice were sacrificed and the tumours removed, measured and weighted. Tumour specimens were formalin fixated and embedded in paraffin for further immunohistochemical analysis.

The animal study was approved by the state agency for Nature, Environment and Consumer Protection NRW (G 208/14) and was carried out in accordance to the EU Directive 2010/63/EU for animal experiments.

### Statistical analysis

Overall survival was defined as the time from the date of surgery until death of any cause. Survivors were censored at the date of the last follow-up. For survival analyses IRS were categorized into high expression (≥median) and low expression (<median). Univariate survival analyses were performed by comparing Kaplan–Meier curves and the log-rank (Mantel Cox) test. For multivariate analyses, Cox regression analyses were used to estimate hazard ratios (HR) with 95% confidence intervals (CI).

To explore statistical differences in the expression levels between survivin and XIAP in accordance to clinicopathological parameters, the non-parametric Mann-Whitney U test and Fisher’s exact test were employed. Cell culture experiments were repeated at least three times and evaluated for statistical significance accordingly. The mean 50% inhibitory concentration (IC_50_) was calculated by logistic analysis. For *in vivo* experiments, differences in tumour size and weight were assessed by the Wilcoxon matched pairs and two-tailed Mann-Whitney U test as indicated.

All statistical analyses were performed using GraphPad Prism (Version 6, GraphPad Software, San Diego, California, USA) or the Statistical Software R version 3.1.0 (R Development Core Team, 2014). A *p*-value < 0.05 was considered to indicate statistical significance.

All data generated or analysed during this study are included in this published article (and its Supplementary Information files).

## Electronic supplementary material

Supplementary Information
