# Supplementary material for: Survivin and XIAP – two potential biological targets in follicular thyroid carcinoma
Source: Sci Rep. 2017 Sep 12;7:11383. doi: 10.1038/s41598-017-11426-3 (PMC5595817; doi:10.1038/s41598-017-11426-3)
Supplement: Supplementary file 1 — Supplementary Information [file 41598_2017_11426_MOESM1_ESM.pdf]

Supplementary Information

Survivin and XIAP – two potential biological targets in follicular thyroid carcinoma

Thomas A. Werner[1], Levent Dizdar[1], Inga Nolten[1], Jasmin C. Riemer[2], Sabrina Mersch[1], Sina C. Schütte[1], Christiane Driemel[1], Pablo E. Verde[3], Katharina Raba[4], Stefan A. Topp[1], Matthias Schott[5], Wolfram T. Knoefel[1], Andreas Krieg[1]

[1]Department of Surgery (A), Heinrich-Heine-University and University Hospital Duesseldorf, Moorenstr. 5, 40225 Duesseldorf, Germany

[2]Institute of Pathology, Heinrich-Heine-University and University Hospital Duesseldorf, Moorenstr. 5, 40225 Duesseldorf, Germany

[3]Coordination Centre for Clinical Trials, Heinrich-Heine-University and University Hospital Duesseldorf, Moorenstr. 5, 40225 Duesseldorf, Germany

[4]Institute for Transplantation Diagnostics and Cell Therapeutics, Heinrich-Heine-University and University Hospital Duesseldorf, Moorenstr. 5, 40225, Duesseldorf, Germany

[5]Division for Specific Endocrinology, Heinrich-Heine-University and University Hospital Duesseldorf, Moorenstr. 5, 40225, Duesseldorf, Germany

Correspondence to: Andreas Krieg, Department of Surgery (A), Heinrich-Heine-University and University Hospital Duesseldorf, Moorenstr. 5, Bldg. 12.46, 40225 Duesseldorf, Germany; Phone: + 49 211 81 19251; Fax: + 49 211 81 19205; Email: andreas.krieg@med.uni-duesseldorf.de

Supplementary Figure 1: Inhibition of survivin and XIAP impairs FTC cell proliferation in vitro. Gene-specific shRNA knockdown of survivin (SVV) and XIAP decreased cell proliferation in both FTC cell lines TT2609-C02 **(a)** and FTC133 **(b)**. Non-specific shRNA served as control (Ctrl). All values are expressed in means + SEM of at least three independent experiments. Statistical significance was calculated by two-tailed nonparametric Mann-Whitney test (**p<0.01, ***p<0.001).

Supplementary Figure 2: Effect of small molecule survivin or XIAP inhibitors on proliferation of FTC cells. FTC cell lines TT2609-C02 and FTC133 were treated with increasing concentrations of **(a)** YM155, **(b)** M4N and **(c)** AT406. Changes in cell proliferation are illustrated in a logarithmic fashion to demonstrate the dose dependent effect of the respective compounds. IC_{50} values represent the mean 50 % inhibitory concentration, where applicable.

Supplementary Figure 3: Full-length western blots.

Blots display the protein expression of **(a)** survivin (SVV) and **(b)** XIAP in the gene-specific shRNA knockdown cell lines SVV KD (survivin knockdown) and XIAP KD (XIAP knockdown) as compared to the non-specific shRNA transfected control cell line (Ctrl). Both FTC cell lines TT2609-C02 and FTC133 were treated with increasing concentrations of YM155, M4N and AT406 and protein expression levels of IAP family members **(c)** survivin, **(d)** XIAP, **(e)** cIAP1 and **(f)** cIAP2 were investigated. Black lanes are inserted where lanes were not directly adjacent on the original blot. The used lanes are labelled by black rectangles. GAPDH served as loading control.

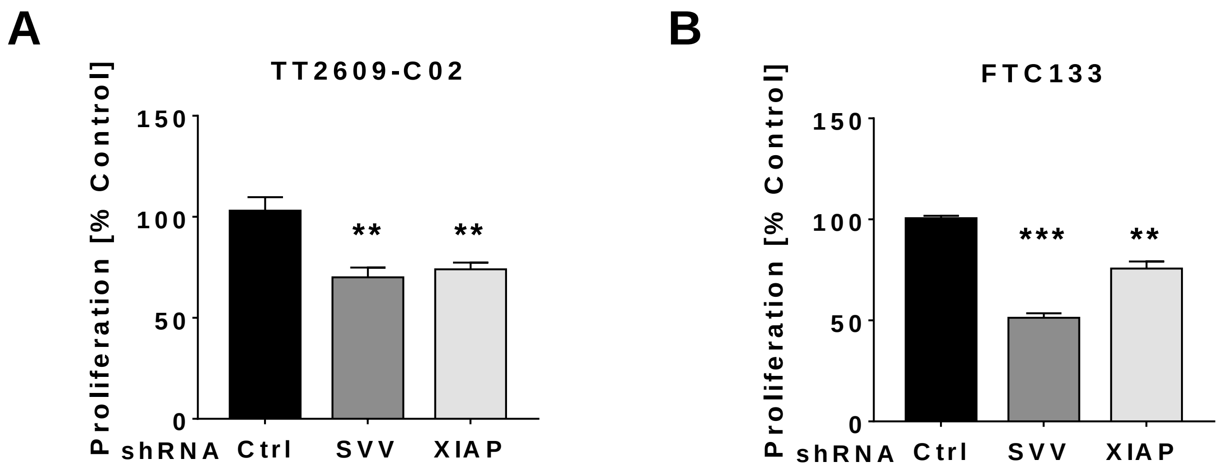

Supplementary Figure 1

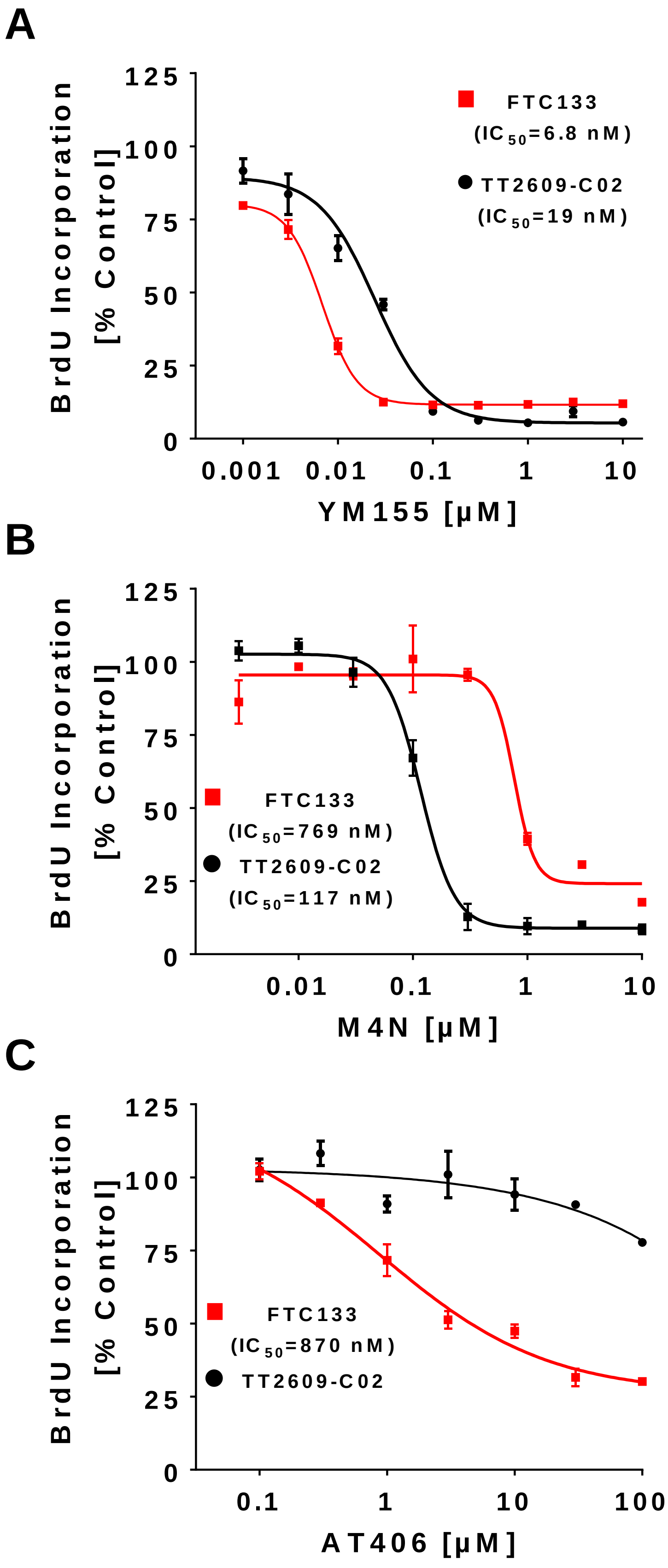

Supplementary Figure 2

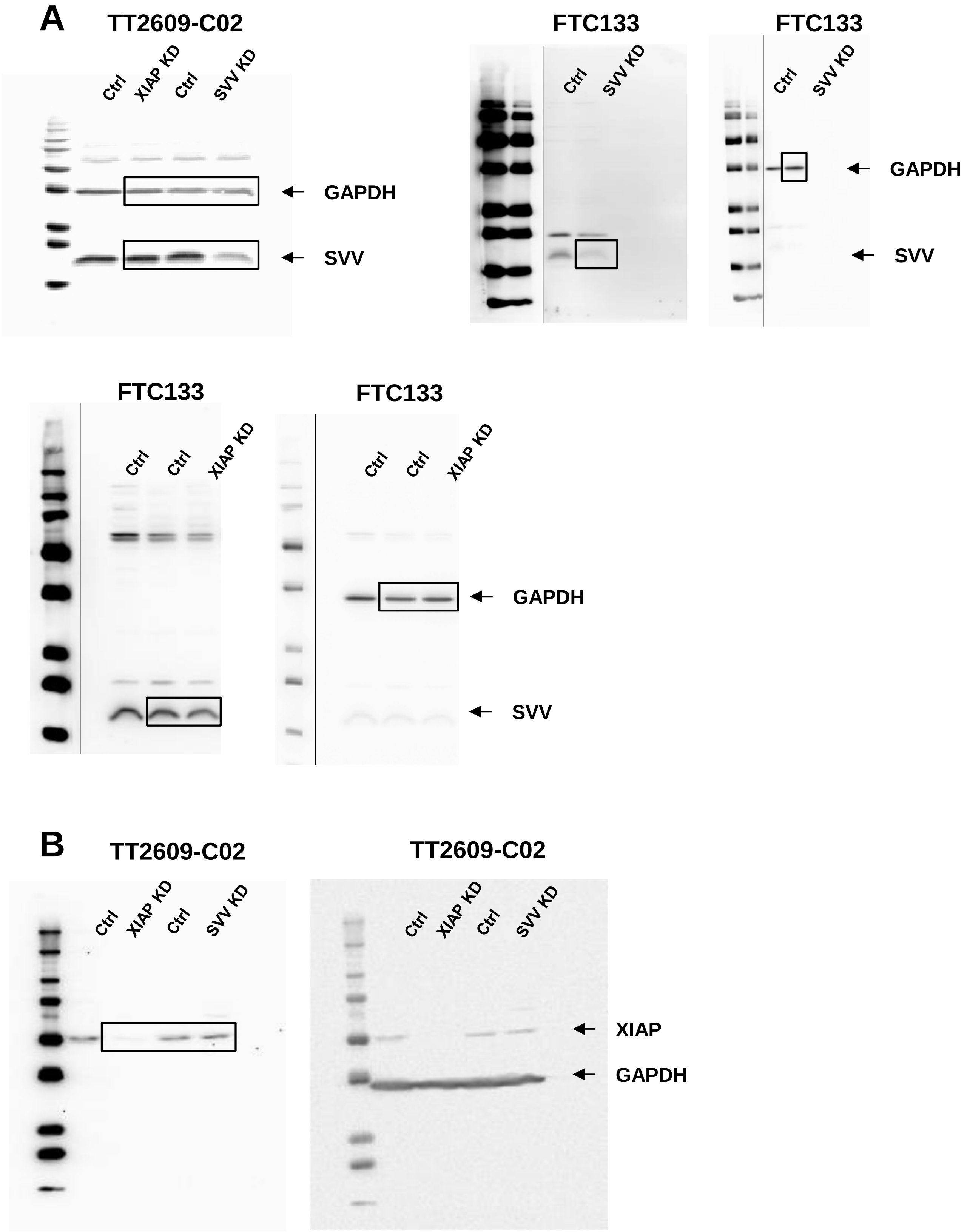
A
TT2609-C02
Ctrl
XIAP KD
Ctrl
SVV KD
GAPDH
SVV
FTC133
Ctrl
SVV KD
FTC133
Ctrl
SVV KD
GAPDH
SVV
FTC133
Ctrl
Ctrl
XIAP KD
FTC133
Ctrl
Ctrl
XIAP KD
GAPDH
SVV
B
TT2609-C02
Ctrl
XIAP KD
Ctrl
SVV KD
TT2609-C02
Ctrl
XIAP KD
Ctrl
SVV KD
XIAP
GAPDH

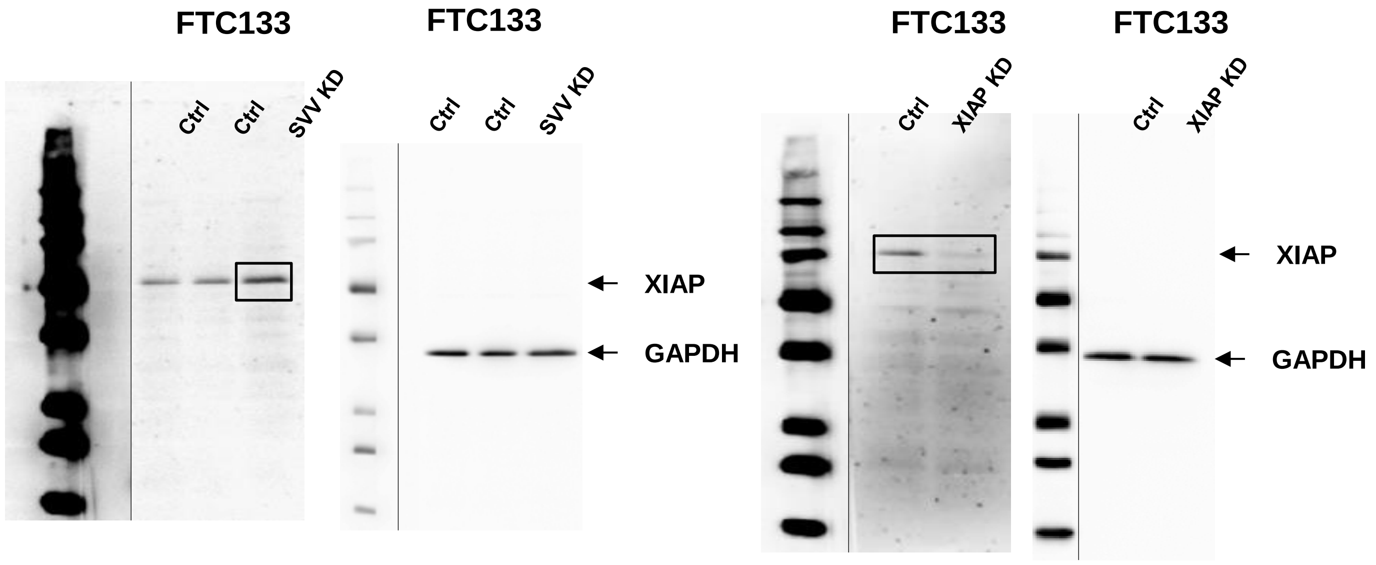
FTC133
Ctrl
Ctrl
SVV KD
FTC133
Ctrl
Ctrl
SVV KD
XIAP
GAPDH
FTC133
Ctrl
XIAP KD
FTC133
Ctrl
XIAP KD
XIAP
GAPDH

C

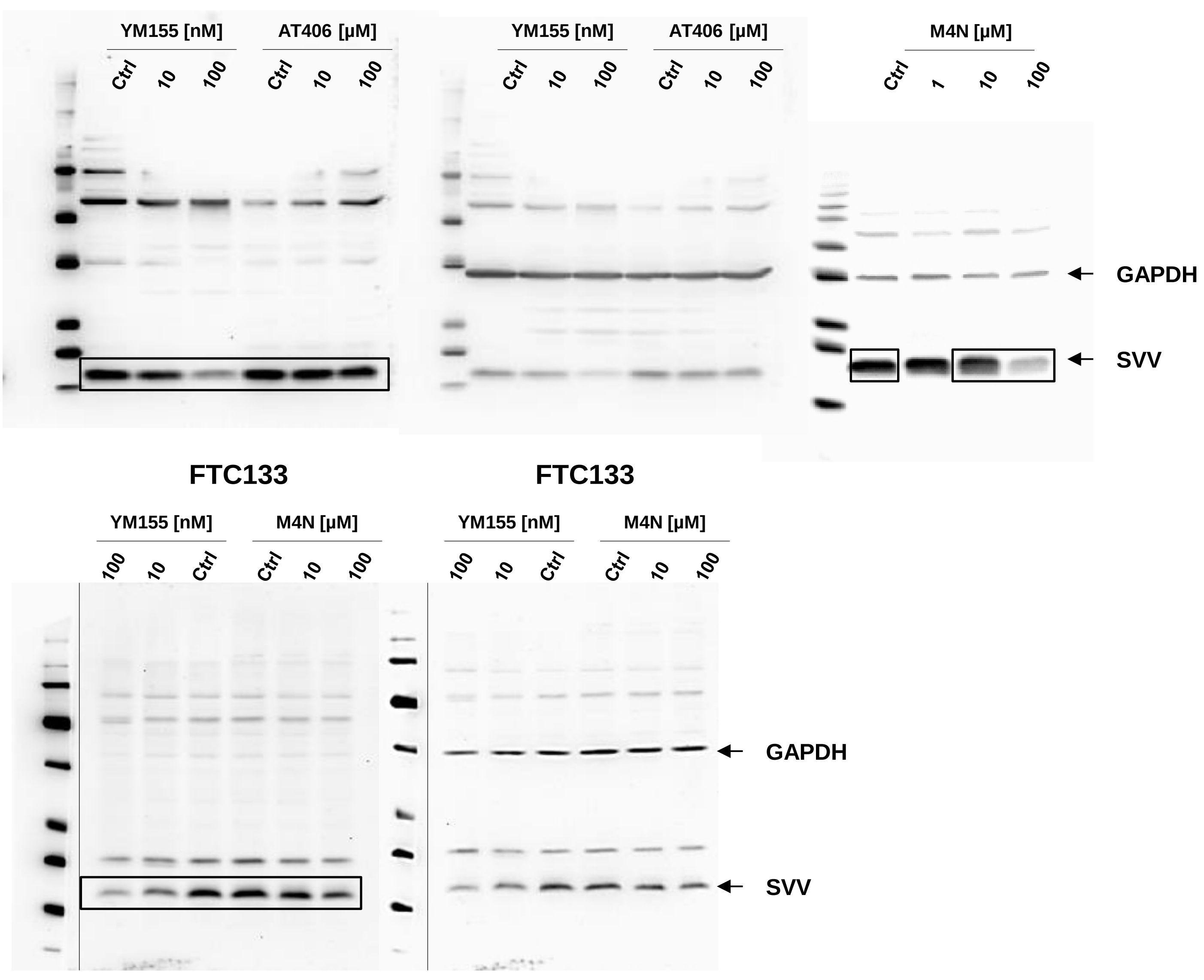
TT2609-C02
YM155 [nM]
AT406 [µM]
Ctrl
10
100
Ctrl
10
100
TT2609-C02
YM155 [nM]
AT406 [µM]
Ctrl
10
100
Ctrl
10
100
TT2609-C02
M4N [µM]
Ctrl
1
10
100
GAPDH
SVV
FTC133
YM155 [nM]
M4N [µM]
100
10
Ctrl
Ctrl
10
100
FTC133
YM155 [nM]
M4N [µM]
100
10
Ctrl
Ctrl
10
100
GAPDH
SVV

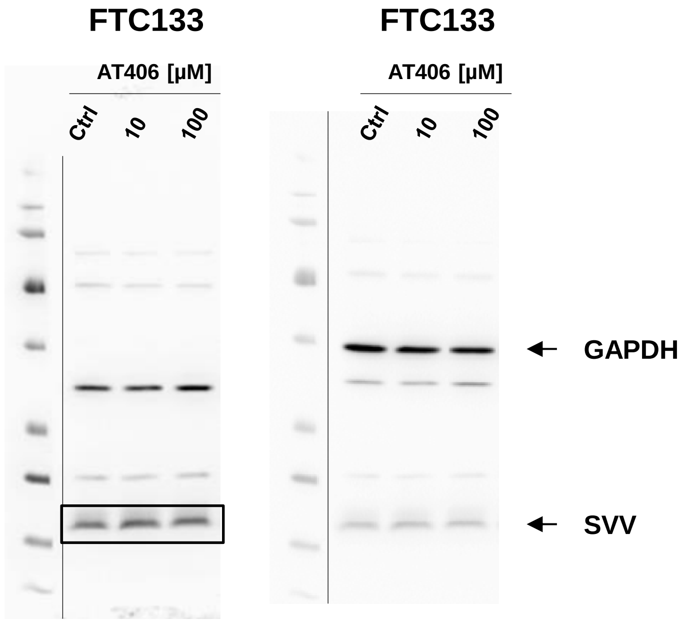
FTC133
AT406 [µM]
Ctrl
10
100
FTC133
AT406 [µM]
Ctrl
10
100
GAPDH
SVV

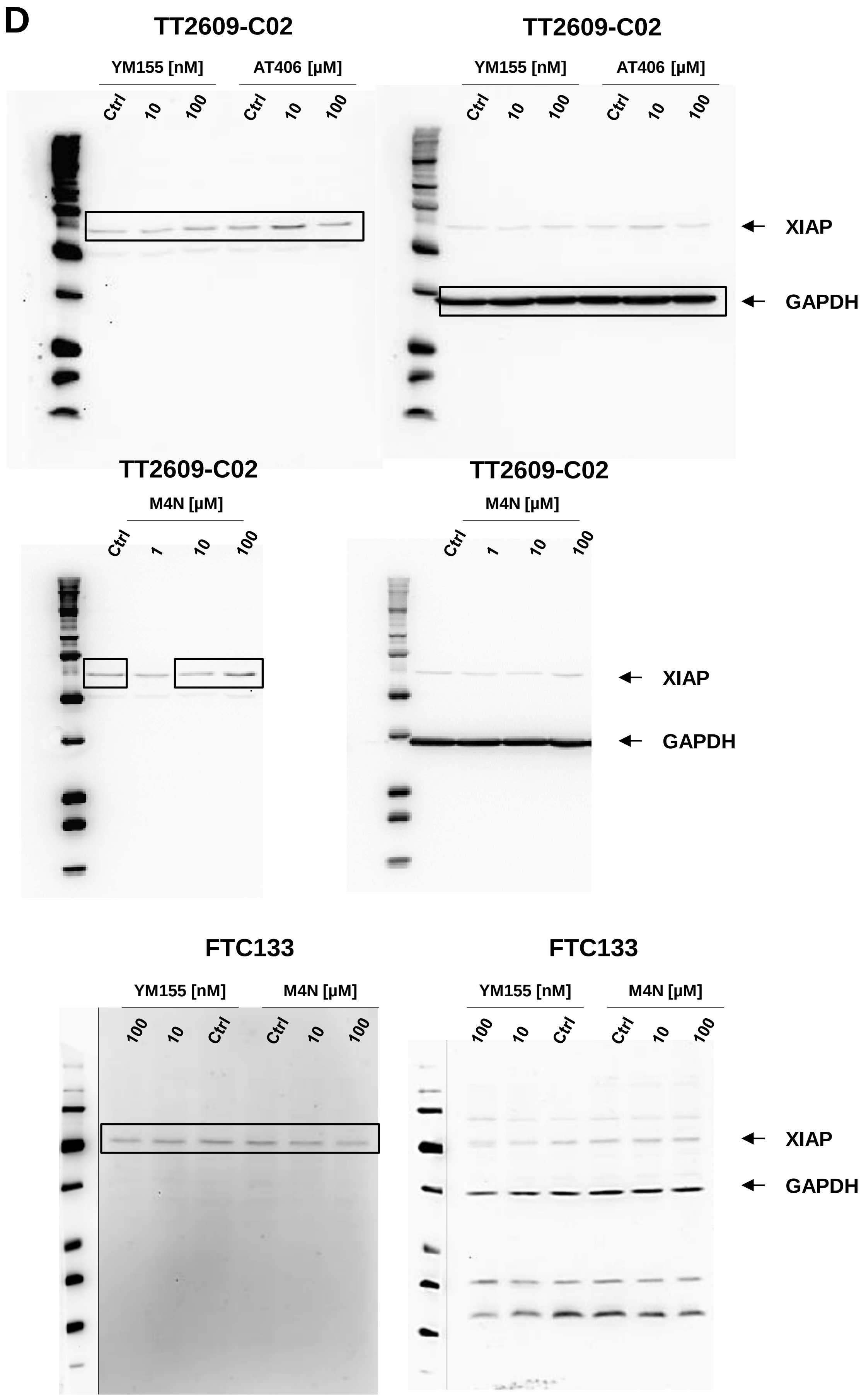
D
TT2609-C02
YM155 [nM]
AT406 [µM]
Ctrl
10
100
Ctrl
10
100
TT2609-C02
YM155 [nM]
AT406 [µM]
Ctrl
10
100
Ctrl
10
100
XIAP
GAPDH
TT2609-C02
M4N [µM]
Ctrl
1
10
100
TT2609-C02
M4N [µM]
Ctrl
1
10
100
XIAP
GAPDH
FTC133
YM155 [nM]
M4N [µM]
100
10
Ctrl
Ctrl
10
100
FTC133
YM155 [nM]
M4N [µM]
100
10
Ctrl
Ctrl
10
100
XIAP
GAPDH

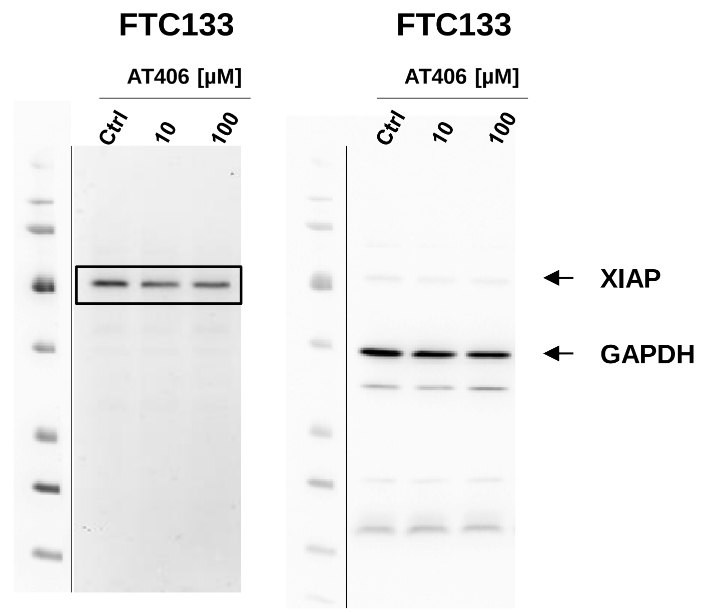
FTC133
AT406 [µM]
Ctrl
10
100
FTC133
AT406 [µM]
Ctrl
10
100
XIAP
GAPDH

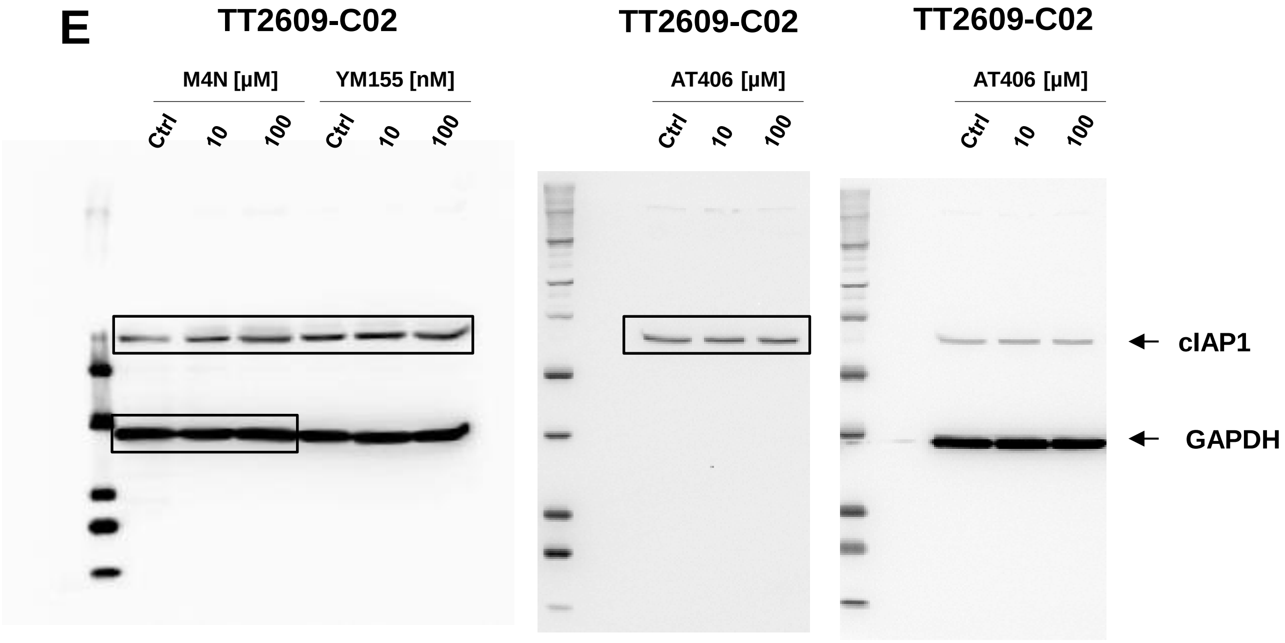
E
TT2609-C02
M4N [µM]
YM155 [nM]
Ctrl
10
100
Ctrl
10
100
TT2609-C02
AT406 [µM]
Ctrl
10
100
TT2609-C02
AT406 [µM]
Ctrl
10
100
cIAP1
GAPDH

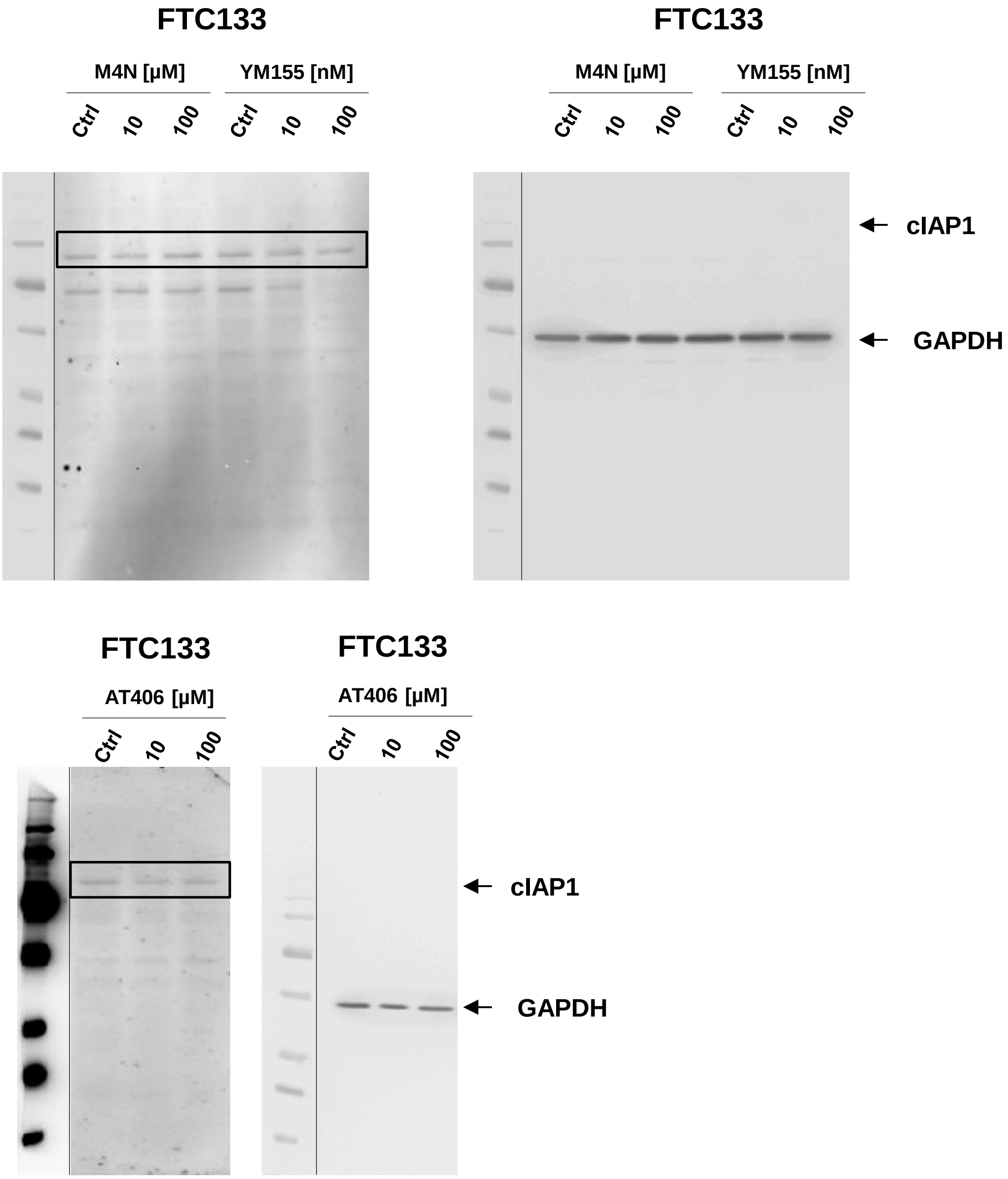
FTC133
M4N [µM]
YM155 [nM]
Ctrl
10
100
Ctrl
10
100
FTC133
M4N [µM]
YM155 [nM]
Ctrl
10
100
Ctrl
10
100
cIAP1
GAPDH
FTC133
AT406 [µM]
Ctrl
10
100
FTC133
AT406 [µM]
Ctrl
10
100
cIAP1
GAPDH

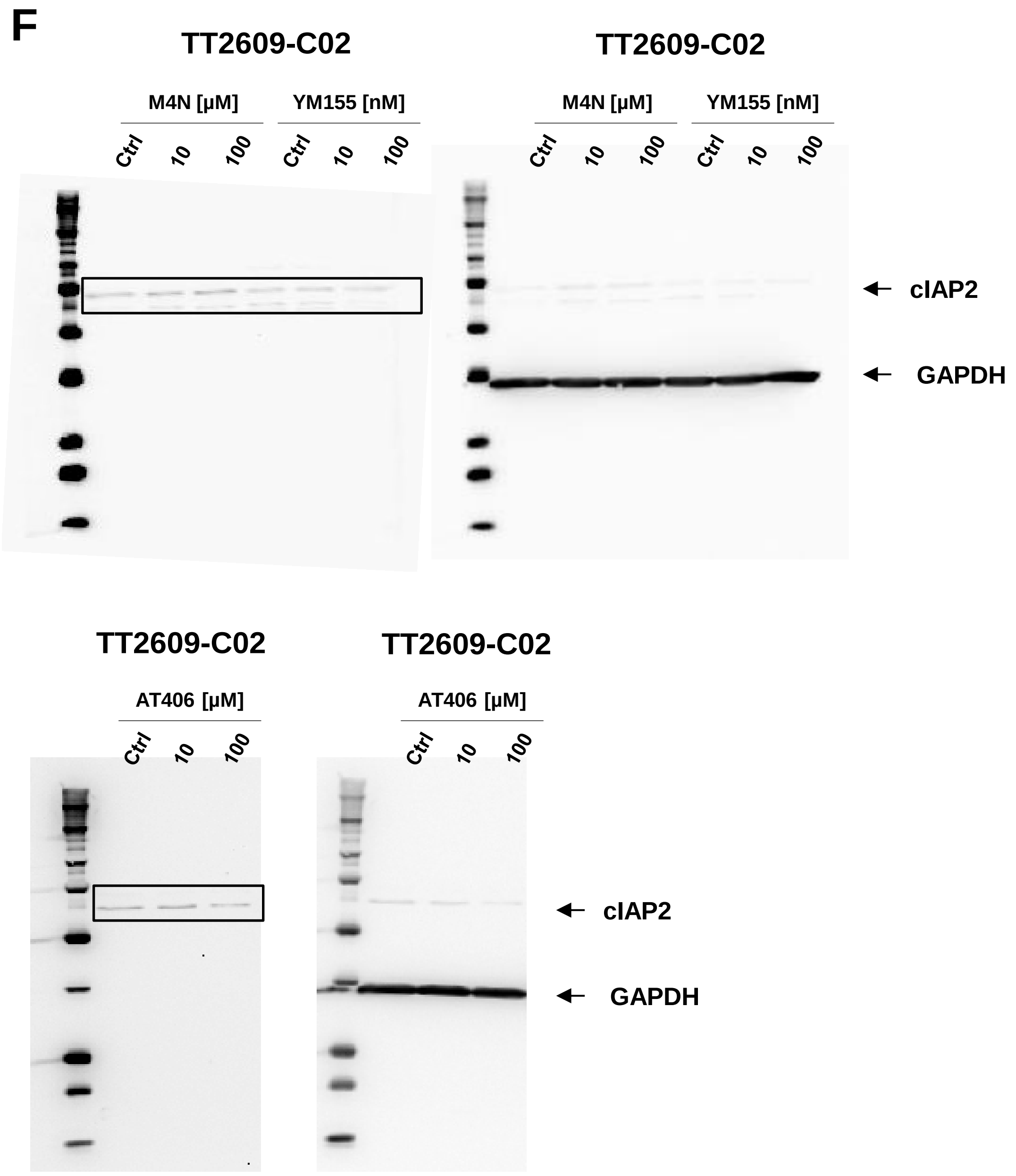
F
TT2609-C02
M4N [µM]
YM155 [nM]
Ctrl
10
100
Ctrl
10
100
TT2609-C02
M4N [µM]
YM155 [nM]
Ctrl
10
100
Ctrl
10
100
cIAP2
GAPDH
TT2609-C02
AT406 [µM]
Ctrl
10
100
TT2609-C02
AT406 [µM]
Ctrl
10
100
cIAP2
GAPDH

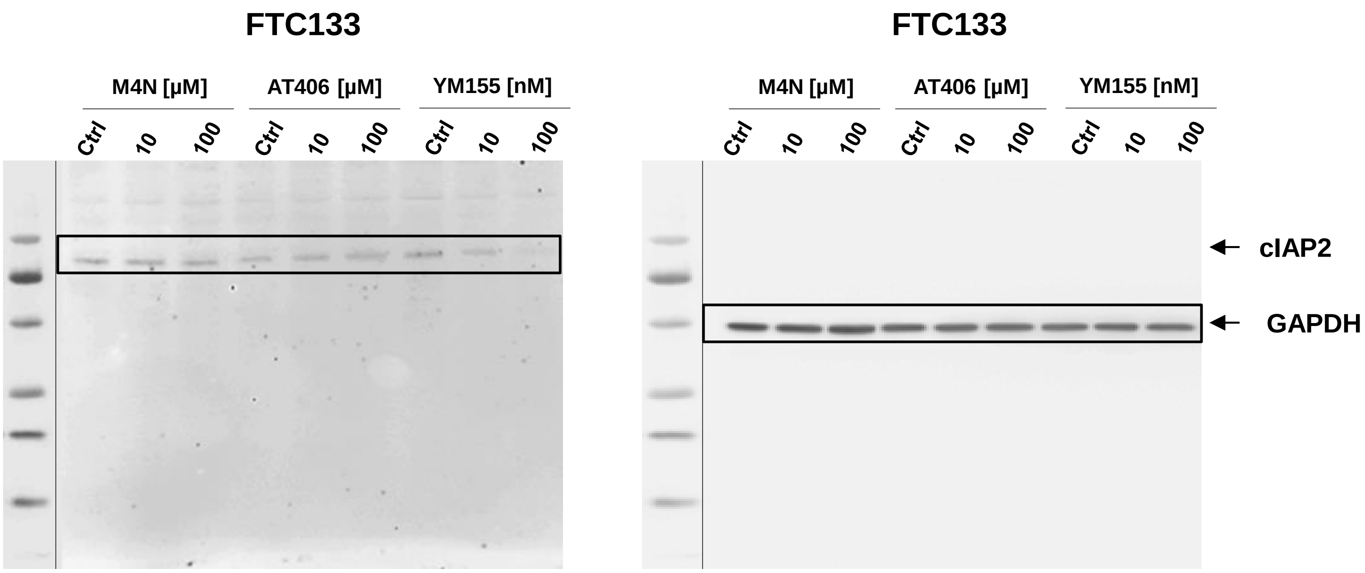

Supplementary Figure 3

Supplementary Table 1: Patient characteristics (n = 44)

Variables	No. of patients (%)
Total	44
**Age**	
Median (range); years	59 (17–85)
**Gender**	
Male	19 (43)
Female	25 (57)
**Type of surgery**	
Hemithyroidectomy	3 (7)
with unilateral ND	1 (2)
Subtotal thyroidectomy	11 (25)
with unilateral ND	4 (9)
with central LND	1 (2)
Total thyroidectomy	7 (16)
with central LND	4 (9)
with unilateral ND	2 (5)
with bilateral ND	11 (25)
**Tumour stage**	
T1/2	24 (55)
T3/4	20 (45)
**Lymph node metastasis**	
N0	41 (93)
N1a/b	3 (7)
**Distant metastasis**	
M0	38 (86)
M1	6 (14)
**UICC stage**	
UICC I/II	22 (50)
UICC III/IV	22 (50)

Abbreviations: ND = neck dissection; LND = lymph node dissection; UICC = Union internationale contre le cancer.

Supplementary Table 2: Correlation between survivin or XIAP and clinicopathological parameters in FTC

	**Survivin expression**			**XIAP expression**		
**Variables**	**Low, n = 14 (%)**	**High, n = 30 (%)**	***p*-value**	**Low, n = 20 (%)**	**High, n = 24 (%)**	***p*-value**
Age, mean ±s.d.	49 ± 14.7	60.5 ± 17.2	0.3319	60 ± 14	53 ± 19	0.7626
**Gender**						
Male	5 (36)	14 (47)		9 (45)	10 (42)	
Female	9 (64)	16 (53)	0.5340	11 (55)	14 (58)	1
**Tumour stage**						
T1/2	13(93)	11 (37)		10 (50)	14 (58)	
T3/4	1 (7)	19 (63)	**0.0008*** **	10 (50)	10 (42)	0.7619
**Lymph node metastasis**						
N0	12 (86)	29 (97)		19 (95)	22 (92)	
N1a/b	2 (14)	1 (3)	0.2336	1 (5)	2 (8)	1
**Distant metastasis**						
M0	14 (100)	24 (80)		18 (90)	20 (83)	
M1	0 (0)	6 (20)	0.1547	2 (10)	4 (17)	0.6731
**UICC stage**						
UICC I/II	11 (79)	11 (37)		10 (50)	12 (50)	
UICC III/IV	3 (21)	19 (63)	**0.0217***	10 (50)	12 (50)	1

Abbreviations: FTC = follicular thyroid carcinoma; XIAP = X-Linked inhibitor of apoptosis protein; UICC = Union internationale contre le cancer; *p<0.05; ***p<0.001.
